# Atypical lymphoid proliferations associated with therapeutic intervention: a report of the 2024 EA4HP/SH lymphoma workshop

**DOI:** 10.1007/s00428-025-04197-0

**Published:** 2025-08-13

**Authors:** Ioannis Anagnostopoulos, Tanja Lakic, Olga Balague, Michiel Van den Brand, Stefan Dirnhofer, Gorana Gasljevic, Camille Laurent, Maurilio Ponzoni, Leticia Quintanilla-Martinez, Birgitta Sander, James R. Cook

**Affiliations:** 1 Institut für Pathologie, Julius-Maximilians Universität, Josef-Schneider-Straße 2, 97080 Würzburg, Germany; 2 Faculty of Medicine Novi Sad, University of Novi Sad and University Clinical Center of Vojvodina, Novi Sad, Serbia; 3 Pathology Department, Hospital Clinic, Barcelona, Spain; 4 Department of Pathology, Radboud University Medical Center, Nijmegen, the Netherlands; 5 Pathology-DNA location Rijnstate Hospital, Arnhem, the Netherlands; 6 Institute of Medical Genetics and Pathology, University Hospital Basel, University of Basel, Basel, Switzerland; 7 Department of Pathology, Institute of Oncology, Ljubljana, Slovenia; 8 Medical Faculty, University of Maribor, Maribor, Slovenia; 9 Department of Pathology, Cancer Institute, Toulouse University Hospital Center, University of Toulouse-Oncopole, Toulouse, France; 10 Ateneo Vita-Salute San Raffaele University, Hematopathology Diagnostic Area, San Raffaele Scientific Institute, Pathology Unit, Milan, Italy; 11 Institut für Pathologie und Neuropathologie, Eberhard-Karls-Universität Tübingen and Comprehensive Cancer Center, University Hospital Tübingen, Tübingen, Germany; 12 Cluster of Excellence iFIT (EXC2180), Image-guided and functionally Instructed Tumor therapies” Eberhard-Karls-University, Tübingen, Germany; 13 Department of Laboratory Medicine, Division of Pathology, Karolinska Institute and Karolinska University Hospital, Stockholm, Sweden; 14 Department of Laboratory Medicine, Cleveland Clinic, Cleveland, OH USA

**Keywords:** Iatrogenic immunodeficiency, DRESS, CAR T-cell therapy, Pseudo-Richter transformation, Dasatinib lymphadenopathy, COVID-19 vaccination

## Abstract

The challenging boundaries between neoplastic and reactive lymphoproliferations were discussed during the 2024 European Association for Haematopathology/Society for Hematopathology workshop in Dubrovnik, Croatia. Session 3 focussed on the atypical lymphoid proliferations associated with therapeutic interventions. Forty-four cases were submitted representing a broad spectrum of lymphoproliferative disorders (LPDs) encountered in the settings of immunosuppressive and immunomodulatory therapies, various interventions for solid tumor treatment, drug reaction with eosinophilia and systemic symptoms (DRESS), CAR T-cell therapy for B-cell lymphomas, Bruton tyrosine kinase inhibitors (BTKI) for SLL/CLL treatment, ABL-kinase inhibitor dasatinib, and COVID-19 vaccination. The cases of this session highlighted the importance of having sufficient clinical information including drug history and distribution of disease in order to achieve reliable diagnosis. Among LPDs associated with immunosuppressive and immunomodulatory therapies, the most challenging were T- and NK-derived infiltrates as they ranged from non-clonal to clonal. DRESS-associated lymphadenopathy exhibited variable histologic patterns with the most difficult differential diagnosis being with a T-cell lymphoma. LPDs observed after CAR T-cell therapy for B-cell neoplasms exhibited unexpected phenotypes resulting either from lineage switching/transdifferentiation, or from harvested T-cells already harbouring cancer-associated variants. Temporary interruption of BTKI treatment for CLL/SLL due to surgical procedures led to a “Pseudo-Richter transformation” that disappeared after reintroduction of therapy. Dasatinib led to a lymphadenopathy with a peculiar florid follicular hyperplasia that regressed after discontinuation of therapy. The findings of the few thoroughly studied COVID-19 vaccination associated lymphadenopathy cases reflected a disordered immune response. This report describes the most important features for diagnosis of these challenging cases.

**Supplementary Information:**

The online version contains supplementary material available at 10.1007/s00428-025-04197-0.

## Introduction

Therapeutic interventions may be associated with a wide range of lymphoproliferative disorders (LPDs). The most widely studied are those arising in the setting of iatrogenic immunosuppression after solid organ or hematopoietic cell transplantation. It is also well known that immunosuppressive and immunomodulatory drugs administered for treatment of autoimmune and rheumatological diseases can affect the patient’s immune system causing an immunodeficiency background. This iatrogenic immunodeficiency may be further aggravated by high patient’s age associated with a variable degree of immunosenescence [1]. In this immunodeficiency setting, a reactivation of latent Epstein-Barr virus (EBV) infection may also occur. These circumstances can lead to the development of a broad and heterogeneous spectrum of LPDs that encompasses a variety of lymphoid hyperplasias (follicular hyperplasia, progressive transformation of germinal centers, Castleman-disease-like changes, infectious mononucleosis-like hyperplasia, plasmacytic hyperplasia, other paracortical hyperplasias), some LPDs leading to an obliteration of underlying tissue architecture (polymorphic LPD, EBV+ mucocutaneous ulcer), as well as low grade and aggressive lymphomas [2]. The spectrum of iatrogenic immunodeficiencies and immune dysregulations is constantly expanding and includes settings of multi-chemotherapy [3, 4], immune checkpoint and targeted regimens for solid cancers [5–7] and hematologic malignancies, chimeric antigen receptor (CAR) T-cell therapy for lymphomas [8], as well as next-generation immunosuppression in autoimmune and inflammatory diseases [9, 10].

The major theme of the 2024 European Association for Haematopathology (EA4HP) and Society for Hematopathology (SH) workshop in Dubrovnik, Croatia, was “Exploring the boundaries between neoplastic and reactive lymphoproliferations”. A session was dedicated to atypical lymphoid proliferations associated with therapeutic intervention. Forty-four cases were submitted representing the challenges in the diagnosis of lymphoproliferations developing in association with the currently constantly expanding spectrum of therapeutic interventions. The cases were divided into the following thematic groups to illustrate not only the diagnostic challenges but also interesting biological features.

1. Lymphoid proliferations associated with immunosuppression or immunomodulatory therapy
2. Lymphoid proliferations associated with interventions for solid tumors
3. Drug reaction with eosinophilia and systemic symptoms (DRESS)
4. CAR T-cell therapy associated complications
5. Complications of SLL/CLL treated by Bruton tyrosine kinase inhibitors
6. Dasatinib associated lymphadenopathy
7. COVID-19 vaccination associated lymphadenopathy
8. Miscellaneous

## Lymphoid proliferations associated with immunosuppression or immunomodulatory therapy

The iatrogenic LPDs include a diverse spectrum of lesions arising in the setting of immunosuppressive or immunomodulatory drugs administered for many different underlying conditions. The most widely studied and best understood forms of iatrogenic LPDs, the post-transplant lymphoproliferative disorders (PTLDs), were specifically excluded from this session. The non-transplant related disorders, sometimes referred to as “other iatrogenic immunodeficiency-associated lymphoproliferative disorders (OIIA-LPD)” [11–14], may be divided into polymorphic versus monomorphic histologic categories, analogous to the classification of PTLDs. Details of the clinicopathologic findings in OIIA-LPD are limited, largely derived from case reports and small series. It is clear; however, that lesions associated with particular therapeutic agents may show specific characteristic findings. For example, LPDs arising in patients with autoimmune diseases (typically rheumatoid arthritis) treated with methotrexate, are most commonly EBV-positive diffuse large B-cell lymphoma (DLBCL), polymorphic B-cell LPDs, or classic Hodgkin lymphomas (CHL) [12, 15, 16]. Patients receiving mycophenolate mofetil, which may be administered in the transplant setting or for autoimmune disease such as myasthenia gravis, are at particular risk for EBV-positive CNS DLBCL [17, 18] and cases of hepatosplenic T-cell lymphoma appear to be increased in patients with Crohn’s disease receiving anti-TNF agents together with thiopurines [13, 19, 20]. Further studies are needed to characterize the clinical spectrum of LPDs that arise in the setting of a rapidly growing list of agents, and the cases submitted to this session offer intriguing insights into these uncommon disorders.

Twelve cases of OIIA-LPD were submitted to this session, including 3 polymorphic B-cell LPDs, 5 cases of monomorphic B-cell LPDs, and 4 cases of T- or NK-cell LPDs. It should be noted that while the 2022 International Consensus Classification (ICC) maintains a separate diagnostic category for PTLDs versus OIIA-LPDs [21], the 5^th^ edition WHO classification has eliminated PTLD as a distinct diagnostic category and instead groups all iatrogenic LPDs under one diagnostic umbrella with a recommended tripartite nomenclature specifying histologic pattern, viral status, and clinical setting [22, 23]. For the purposes of this workshop, all cases were classified using diagnostic criteria of both systems.

**Fig. 1:**
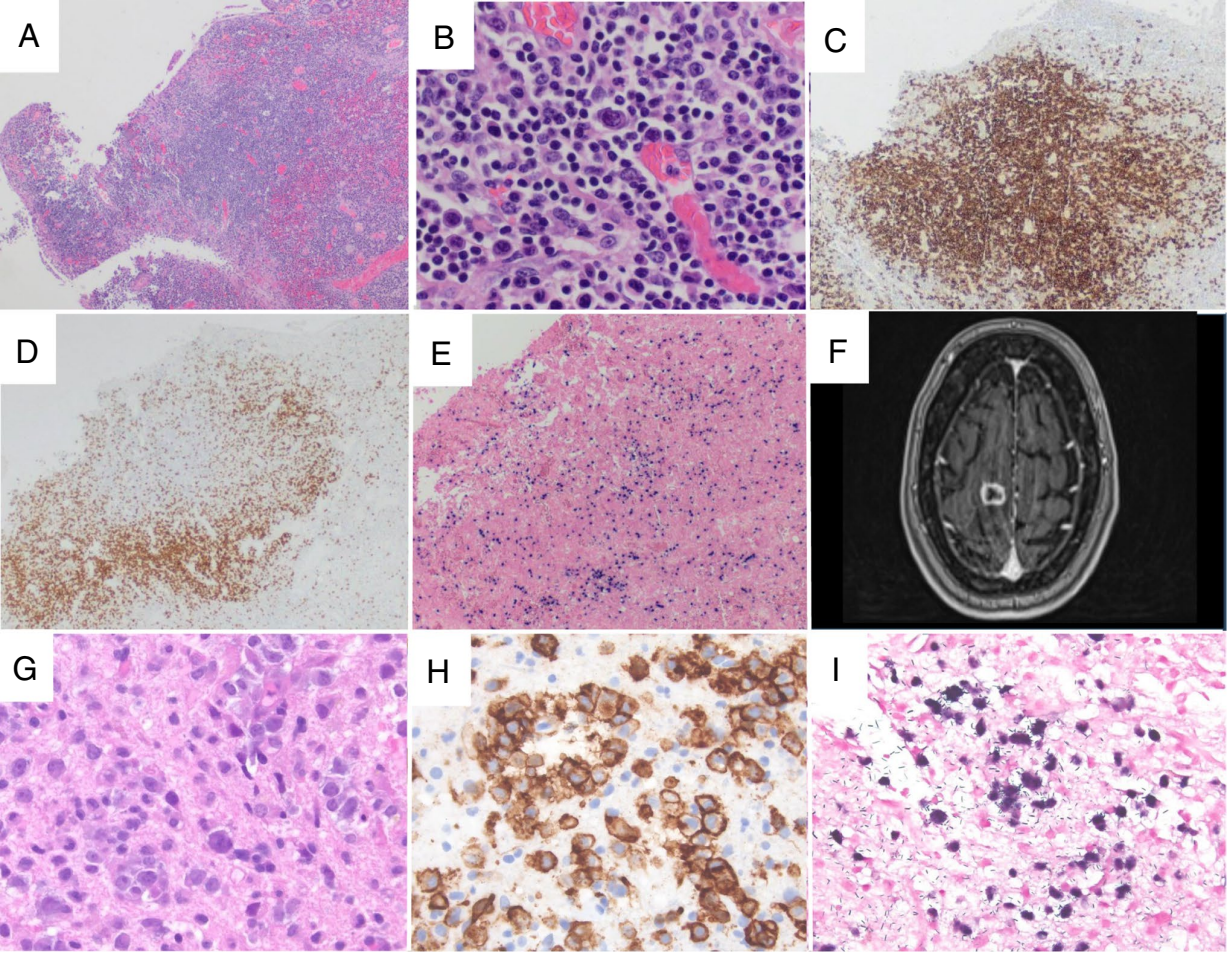
Iatrogenic B-cell lymphoproliferative disorders. Case LYWS-87 (images courtesy of A. Sierra) showed multiple colonic lesions (**A**) with a heterogeneous infiltrate of small lymphocytes, immunoblasts, and scattered large atypical cells (**B**). The infiltrate contained numerous CD20 positive cells (**C**) with a well-defined rim of small CD3 positive cells at the base (**D**). An EBER in situ hybridization stain highlighted scattered positive Reed-Sternberg-like cells (**E**). In case LYWS-359 (images courtesy of L. Carrillo), a patient receiving mycophenolate and hydroxychloroquine for mixed connective tissue disease developed a CNS mass (**F**) which was composed of sheets of large atypical cells (**G**) expressing CD20 (**H**). An EBER in situ hybridization stain was diffusely positive (**I**)

The 3 cases of polymorphic B-cell LPDs (Supplemental Table 1) were each EBV-positive and each were being treated with steroids for autoimmune disease. While some studies have reported an increased risk of lymphoma in patients receiving corticosteroids [24, 25], the precise role of steroid therapy in development of an LPD remains uncertain and difficult to separate from risks that may be associated with the underlying autoimmune disorder. Case LYWS-87 submitted by A. Sierra was a 21-year-old male with ulcerative colitis (UC), receiving vedolizumab and prednisone, who had a long history of poor response to other immunosuppressants. The patient developed ulcerated lesions of the colon, requiring subtotal colectomy, without adenopathy or other mass lesions. The colon contained multiple discrete ulcers with a heterogeneous infiltrate of small B- and T- lymphocytes, polytypic plasma cells, immunoblasts and scattered large atypical EBV-positive B-cells (Figure 1 A–E). Histologic classification of this lesion differed in the ICC and WHO5 systems. While each individual ulcer had features compatible with a diagnosis of an EBV-positive mucocutaneous ulcer (EBV-MCU), the diagnosis of EBV-MCU is restricted to solitary lesions in the ICC and the preceding WHO4R system, and the findings were therefore classified as a polymorphic EBV-positive B-cell LPD. The WHO5, in contrast, allows for a diagnosis of EBV-MCU with multiple lesions within one organ system. This patient was treated with reduction of immunosuppression and remained in complete remission. Cases LYWS-323 from G. Lukose and LYWS-453 from M. B. Pereira each presented with adenopathy and lymph node biopsy disclosed a polymorphic background with scattered EBV-positive cells, which were Reed-Sternberg like (LYWS-323) or of centroblastic morphology (LYWS-453). Clinical follow-up was not available in these latter two cases.

Of 5 cases of monomorphic B-cell OIIA-LPD (Supplemental Table 2), 2 were EBV-positive DLBCL (EBV+ DLBCL) with a diffuse proliferation of large EBV-positive B-cells with a non-germinal center phenotype. Case LYWS-474 submitted by S. Bhavsar was a patient receiving azathioprine for autoimmune hepatitis, who developed an EBV+ DLBCL involving liver, lung, bone, and mediastinal lymph nodes, while LYWS-359 submitted by Louis Carrillo was a CNS EBV+ DLBCL in a patient receiving mycophenolate and hydroxychloroquine for mixed connective tissue disease (Figure 1 F–I). Azathioprine and mycophenolate have been each used for many years as immunosuppressant medications for solid organ transplants and for treatment of autoimmune disorders and carry known risks of iatrogenic LPDs [11, 12, 18]. Case LYWS-379 presented by B. Shah was a patient with a 10-year history of chronic plaque psoriasis, who developed an EBV+ CHL within several months of starting guselkumab, an anti-IL-23 monoclonal antibody. The data available to date suggest that guselkumab is not associated with an increased risk of lymphoma [26], but patients with plaque psoriasis are at an increased risk of lymphoma overall including cutaneous T-cell lymphomas and CHL [27]. The role of other prior immunosuppressive therapies in contributing to the development of this lymphoma, if any, is uncertain. This case illustrates the difficulty in determining the role of a specific agent in promoting lymphomagenesis, especially for relatively new agents with limited data. LYWS-388 from S. Tian was an EBV+ DLBCL arising in the setting of a prior grade 3 A follicular lymphoma with focal EBV-positive areas. It is possible that the CHOP therapy administered in this case contributed to the eventual emergence of the EBV+ DLBCL, but insufficient materials were available for the panel to completely assess the relationships between these samples. Finally, case LYWS-234 submitted by G. Parecki was a patient receiving methotrexate on-and-off for 10 years for eosinophilic granulomatosis with polyangiitis and asthma who developed a pulmonary marginal zone lymphoma. EBER studies demonstrated few scattered positive cells, consistent with background EBV reactivation. It should be noted that diffuse EBV positivity can be seen in rare extranodal marginal zone lymphomas as a PTLD or OIIA-LPD [28, 29], but this is distinct from the few, scattered EBER positive cells that characterize EBV reactivation. Distinguishing background EBV reactivation from a truly EBV positive lymphoma or polymorphic EBV+ LPD may be important for clinical management.

**Fig. 2:**
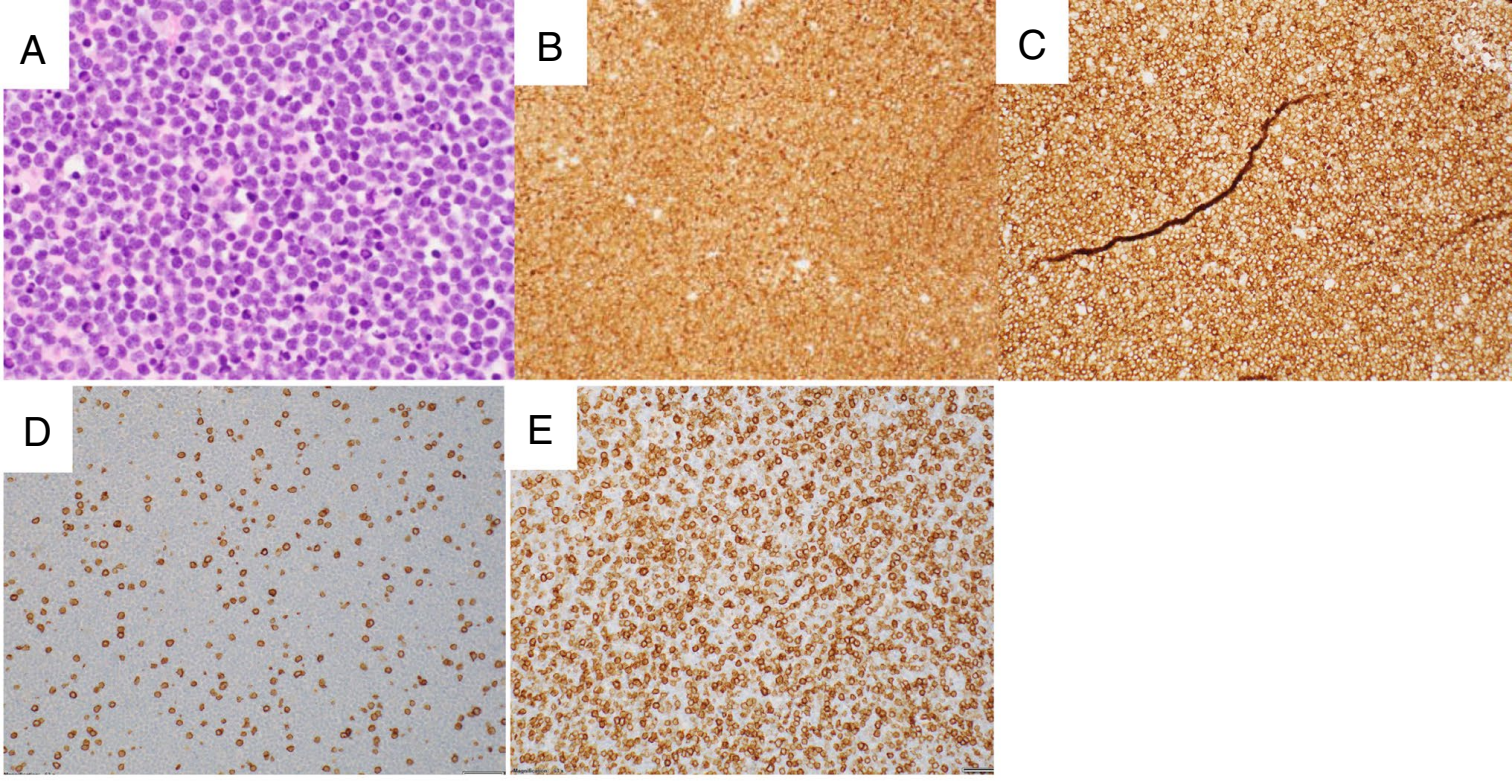
Iatrogenic T-cell lymphoproliferative disorder. In case LYWS-473 (images courtesy of X. Wu), ascites fluid and HLH developed in a patient with a history of CLL and Richter’s transformation placed on everolimus. The cytology cell block showed a monomorphic population of small lymphocytes (**A**), which were positive for CD3 (**B**) and PD1 (**C**). The T-cells included few CD4 positive cells (**D**) with numerous CD8 positive cells (**E**)

Four submitted cases represented EBV-negative T-cell or NK-cell proliferations arising in patients receiving immunosuppressive or immunomodulatory therapy (Supplemental Table 3). Case LYWS-124 from R. L. King was an EBV+ DLBCL arising in a patient receiving multiple immunosuppressive therapies for Crohn’s disease. Following R-CHOP treatment, the patient was diagnosed with an EBV- peripheral T-cell lymphoma, not otherwise specified (PTCL, NOS). PCR studies of both samples showed that the PTCL T-cell clone was not detectable in the EBV+ DLBCL sample, arguing that these represent independent processes and raising the possibility that chemotherapy may have contributed to development of the PTCL. The three remaining cases were examples of unusual T-cell or NK-cell proliferations arising in the setting of etanercept, vedolizumab, and everolimus therapies (LYWS-193, LYWS-287, and LYWS-473, respectively). Case LYWS-193 from J. Sidhu was a patient with rheumatoid arthritis treated with methotrexate and etanercept, a TNF inhibitor. Within 2 months of beginning etanercept, the patient developed severe influenza A with adenopathy, and lymph node biopsy showed a somewhat depleted lymph node with a monotonous infiltrate of small CD4+ T-cells that coexpressed PD1, CD57, and CD200 but were negative for other TFH markers. As this non-canonical-TFH proliferation was not clonal by PCR, the panel favored a diagnosis of an atypical T-cell proliferation rather than an overt lymphoma and the patient died soon after biopsy. Case LYWS-287 submitted by L. M. R. Gjerdrum was a 77-year-old male receiving vedolizumab, an α4β7 integrin antibody for Crohn’s disease, who developed a chronic NK-cell lymphoproliferative disorder (absolute lymphocyte count 5–10 × 10^9^/L). NGS studies showed no *STAT3* mutations, but mutations in *TET2* and *TNFAIP3* were present, and it was difficult to determine if these represented somatic mutations in the lymphocytes versus background clonal myeloid elements. In case LYWS-473 submitted by X. Wu, a 79-year-old man with a history of CLL with CHL-type Richter transformation treated with multiple chemotherapy regimens was placed on everolimus and soon developed pancytopenia, hemophagocytic lymphohistiocytosis (HLH), and ascites. A cytology cell block of the ascites fluid showed a proliferation of monomorphic small lymphocytes that were predominantly CD8 positive with PD1 expression (Figure 2). Despite the presence of a clonal population by PCR, the review panel favored a diagnosis of an atypical clonal T-cell proliferation rather than lymphoma. PD1 expression is characteristic of exhausted cytotoxic T-cell proliferations [30–32], but it is difficult to determine whether this proliferation was secondary to the lymphoma, HLH, or therapy, or any combination thereof. These cases illustrate the complexity of attempting to determine the contribution of these therapeutic agents to T- or NK-cell proliferations, especially for newer therapeutic agents where relatively little data exists in the literature.

## Lymphoid proliferations associated with interventions for solid tumors

Five cases submitted to this session represented LPDs arising in patients being treated for solid tumors (Supplemental Table 4). LYWS-23 submitted by T. Tousseyn was a patient with *EGFR* mutated adenocarcinoma of the lung, who was treated with chemotherapy and an anti-EGFR agent and developed a T-cell lymphoma with TFH phenotype and only focal scattered EBV positive cells, and LYWS-173 from N. Demko was a patient with esophageal adenocarcinoma, who developed an EBV+ polymorphic LPD arising after chemotherapy. Chemotherapy for solid tumors is increasingly recognized as contributing to development of an iatrogenic LPD [3, 4], although the clinical history should be thoroughly examined for any coexisting immunodeficiency, autoimmune disorder, or immunosuppressive medications, which may also promote development of an LPD. The EBV+ polymorphic B-cell LPD in case LYWS-173 was similar to lesions typically arising in the setting of immunosuppression. The T-cell lymphoma in case LYWS-23, in contrast, is largely similar to that which might arise in an immunocompetent host, and it is difficult in such cases to determine to what extent prior chemotherapy truly promoted the development of the lymphoma.

Two cases were examples of LPDs arising in patients treated with anti-PD1 checkpoint inhibitors. Only rare examples of clonal lymphoproliferative disorders have been reported in patients with checkpoint inhibitor therapy [7], which are generally thought to act by increasing immunocompetence via restoring activity of exhausted T-cells [33, 34]. Case LYWS-375 submitted by I. Anagnostopoulos was an EBV+ polymorphic B-cell LPD in a patient receiving pembrolizumab for colorectal carcinoma with microsatellite instability (MSI) due to deficient DNA mismatch repair. LYWS-286 from A. Szumera-Cieckiewicz was a patient receiving nivolumab for *BRAF* mutated melanoma who developed subcutaneous masses. Biopsy showed an EBV negative lymphoid proliferation with morphologic and phenotypic features consistent with a diagnosis of a subcutaneous panniculitis-like T-cell lymphoma. However, cases of panniculitis-like infiltrates have also been reported as immune-related adverse events in patients receiving checkpoint inhibitors [35, 36], and there was insufficient material for molecular studies in this case to confirm T-cell clonality, such that the review panel was unable to render a definitive diagnosis.

**Fig. 3:**
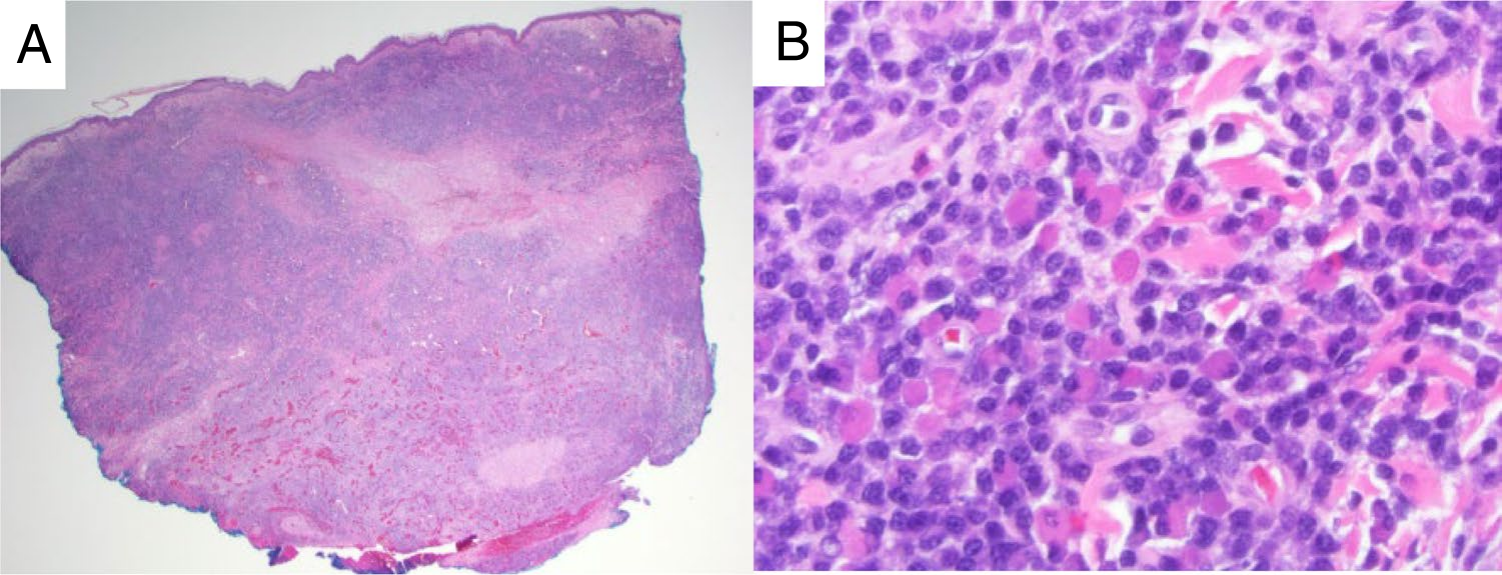
Iatrogenic lymphoproliferative disorder following treatment for solid tumor. Case LYWS-423 (images courtesy of M. Merzianu) was a patient with melanoma who received intralesional talimogene laherparepvec. Follow-up biopsy showed a diffuse infiltrate (**A**) composed of mature plasma cells (**B**) with monotypic kappa light chain expression

Finally, case LYWS-423 submitted by M. Merzianu was a 96-year-old man with a 15-year history of multiply recurrent melanoma as well as a parotid area follicular lymphoma, which had been diagnosed on fine needle aspirate and had not been treated. The melanoma was treated with intralesional talimogene laherparepvec (T-VEC), a modified herpes simplex virus intended to infect and lyse melanoma cells, stimulating an anti-tumoral immune response. A biopsy performed to assess response after 6 weeks (Figure 3) showed no evidence of melanoma, but diffuse sheets of kappa monotypic plasma cells were present with a normal (CD19 positive, CD56 negative) phenotype. PCR studies showed a clonal B-cell receptor rearrangement with clonal amplicons that did not match the prior follicular lymphoma. The pattern of findings suggested a clonal but reactive plasmacytic proliferation secondary to T-VEC therapy, possibly involved in treatment response [37].

## Drug reaction with eosinophilia and systemic symptoms (DRESS)

Drug reaction with eosinophilia and systemic symptoms (DRESS) is a form of severe cutaneous delayed-type hypersensitivity drug reaction that is often characterized by lymphadenopathy and eosinophilia that may raise concern for a lymphoproliferative disorder [38–41]. DRESS is uncommon overall but has been reported to represent up to 23% of cutaneous drug reactions in hospitalized patients, and mortality rates in the US have been estimated to be approximately 5%. The diagnosis of DRESS is based on a pattern of clinical, laboratory, and pathologic findings with different organizations proposing varyingly rigorous sets of diagnostic criteria (Table 1) [40–42]. Antibiotics represent the most common category of inciting drugs, although a very wide range of agents have been implicated including anticonvulsants, anti-inflammatory agents, antivirals, and iodine containing contrast medium, among many others. In addition to hematologic abnormalities that may include eosinophilia and/or atypical lymphocytosis, systemic symptoms may include dysfunction of the liver, kidneys, lung, or heart, although almost every organ system has been reported to be involved [38, 39]. Systemic symptoms may be severe, or even fatal due to organ failure. HHV6 reactivation has been associated with DRESS [38, 39, 43], although the role of HHV6 in the development of DRESS remains controversial and HHV6 is not detectable in all cases. HHV6 reactivation defines typical DRESS using Japanese consensus criteria but it is not required for a diagnosis of DRESS in other systems. Treatment typically consists of withdrawal of the offending agent and steroid therapy with a prolonged taper to decrease the risk of relapse.

**Table 1:** Diagnostic criteria in the initial report of DRESS by Bouquet et al., and criteria employed by subsequent investigators

**Bocquet et al.** [40]	**Japanese Consensus Group** [42]	**RegiSCAR** [41]
1. A cutaneous drug eruption	1. Maculopapular eruption developing >3 weeks after starting a limited number of drugs	1. Acute skin eruption
2. Systemic involvement by lymphadenopathy ≥2 cm in diameter or hepatitis (transaminase ≥2 times upper limit of normal) or interstitial nephritis or interstitial pneumonitis or carditis	2. Prolonged clinical manifestations 2 weeks after discontinuation of the causative drug	2. Fever (>38 °C)
3. Hematologic abnormalities: eosinophilia ≥1.5×10^9^/L or presence of atypical lymphocytes	3. Fever (>38 °C)	3. Lymphadenopathy at ≥2 sites
	4. Liver abnormalities (alanine aminotransferase >100 U/L)	4. Involvement of at least one internal organ
	5. Leukocyte abnormalities (at least 1 present): Leukocytosis (>11×109/L) Atypical lymphocytosis (>5%) Eosinophilia (>1.5×109/L)	5. Lymphocytosis or lymphocytopenia
	6. Lymphadenopathy	6. Peripheral eosinophilia
	7. HHV-6 reactivation	7. Thrombocytopenia
Required: All 3 elements	Required: All criteria (typical) or the first 5 (atypical)	Required: At least 3. A scoring system is also applied to classify degree of certainty.

**Fig. 4:**
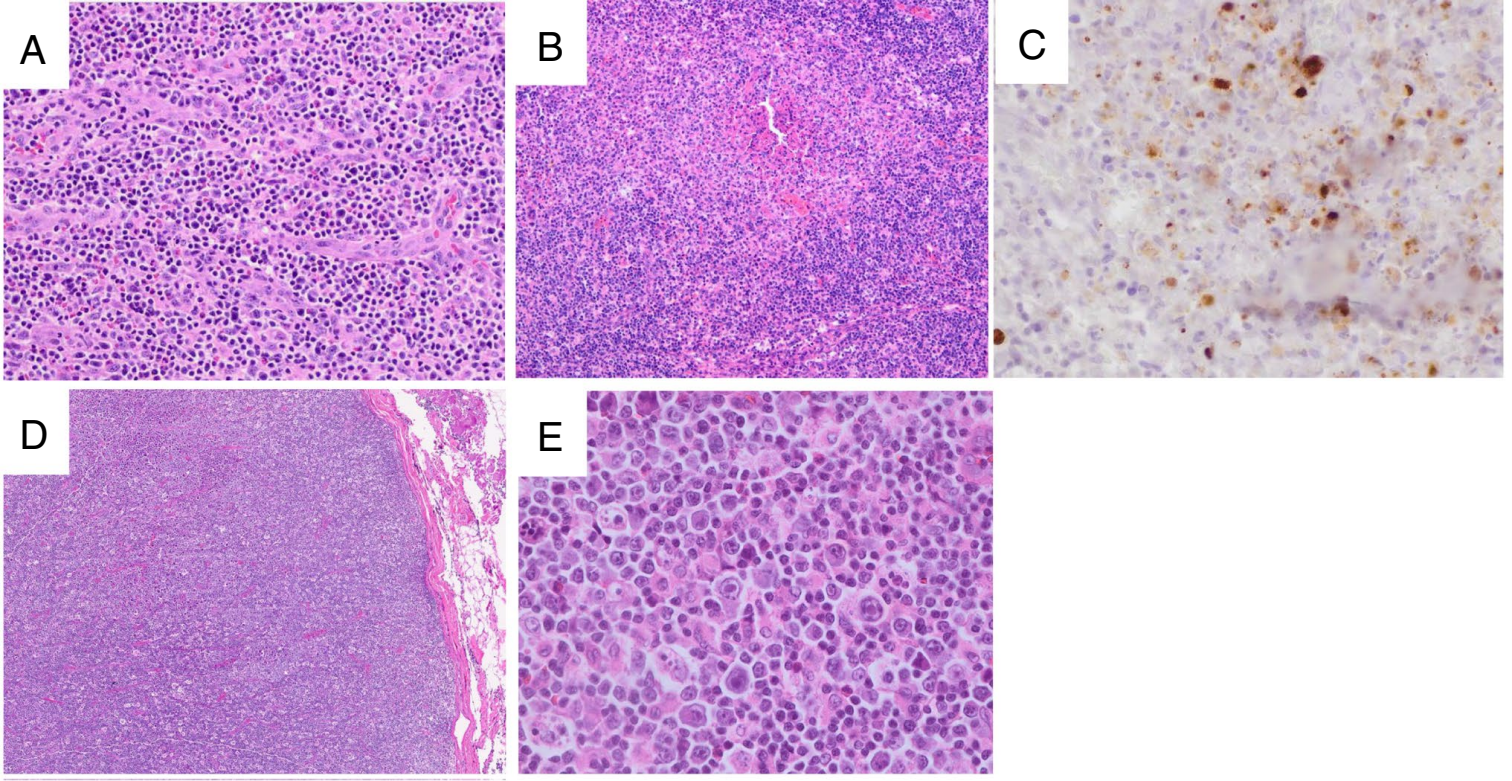
Patterns of lymph node histology in DRESS syndrome. Case LYWS-297 (images courtesy of J.R. Cook) showed an angioimmunoblastic T-cell lymphoma-like pattern with architectural effacement, vascular proliferation, and a proliferation of small lymphocytes and occasional larger cells (**A**). Case LYWS-203 (images courtesy of A. Saglam) exhibited a Kikuchi-like pattern with areas of neutrophil-poor necrosis (**B**) and HHV6 positive cells detected by immunohistochemistry (**C**). A reactive paracortical hyperplasia-like pattern was illustrated in LYWS-208 (images courtesy of A. Flaifel) with expanded paracortical regions (**D**) containing small lymphocytes with scattered large transformed cells (**E**)

Six cases of DRESS were submitted to this session (Supplemental Table 5). Implicated drugs included antibiotics in 4 cases while one patient received doxazosin and bendroflumethiazide (LYWS-32 from D. Pisani) and one received a combination of thalidomide, sulfasalazine, and traditional Chinese medicine (LYWS-86 submitted by Y. Li). Lymph node biopsies were performed in 5/6 cases, highlighting the propensity of DRESS to raise clinical concern for a lymphoproliferative disorder. Four different histologic patterns have been described in DRESS lymph nodes, three of which were illustrated in the submitted cases (Figure 4): reactive paracortical hyperplasia (LYWS-86 from Yu Li and LYWS-208 from J. V. Alves De Castro), Kikuchi-like (LYWS-203 submitted by A. Saglam), angioimmunoblastic T-cell lymphoma-like (LYWS-107 submitted by S. El Hussein and LYWS-297 submitted by J. R. Cook), and Hodgkin-like (not identified in this cohort). All cases were reported to show retention of the pan-T-cell-antigens studied, and PCR studies for T-cell clonality were performed in three cases, with polyclonal results in each case. Immunohistochemistry for HHV6 was performed by the submitter of LYWS-208 and by the review panel in additional three cases, and 2/4 (50%) were positive.

In light of the potentially fatal complications of DRESS, timely recognition of this diagnosis is critical for patient management [38, 39]. The heterogeneity of lymph node morphology makes the correct diagnosis challenging, and some cases may raise concerns for a T-cell neoplasm, especially TFH lymphoma. PCR studies were polyclonal in all cases studied, which may provide some reassurance in routine practice, but ultimately establishing the correct diagnosis requires correlation with the clinical findings and the time course since initiation of drug therapy.

## CAR T-cell therapy associated complications

Adoptive cellular therapy using CARs has altered the landscape of cancer immunotherapy. CAR T-cells are genetically modified autologous T-cells manufactured to express a CAR gene. The efficacy of CAR T-cells has been proven in patients with relapsed/refractory B-cell derived malignancies, including acute lymphoblastic leukemia (B-ALL) [44], mantle cell lymphoma (MCL) [45], multiple myeloma (MM) [46], and different entities of large B-cell lymphoma (LBCL) [47]. Despite the remarkable therapeutic efficacy of CAR T-cell therapies, there are several known complications. Besides those associated with the direct receipt of CAR T-cells or from the cytotoxic and immunosuppressive regimens that accompany such a treatment like cytokine release syndrome, neurotoxicity syndrome, cytopenias and infections, there are complications requiring haematopathological expertise for clarification of therapy resistance and for characterization of post-infusion malignancies. In addition, pathology laboratories are expected to play a role in monitoring the phenotype, persistence, and tumour infiltration properties of CAR T-cells and the tumour microenvironment for factors that might predict CAR T-cell therapy success. In the workshop, five cases were submitted dealing with CAR T-cell therapy complications: two cases associated with CAR T-cell resistance (LYWS-88 and LYWS-262), and three cases with neoplastic and non-neoplastic T-cell proliferations (LYWS-172, LYWS-186, and LYWS-93).

### Resistance to CAR T-cell therapy

Despite frequent durable remissions induced by this biologically active immunotherapy in haematologic malignancies, over half of the patients show a lack of response or eventually relapse [48] [49, 50]. Available evidence suggests that resistance to CAR T-cells is multifactorial and influenced by factors related not only to the target but also to the effector cells. Antigen loss in tumor cells is one of the major mechanisms of resistance and can occur through various mechanisms, such as gene mutations, deletions, rearrangements, epigenetic modifications, lineage switching also referred to as transdifferentiation, in which sequential neoplasms with apparent different lineages exhibit evidence of derivation from a common ancestor clone, and trogocytosis, in which CAR T-cells gnaw away antigen from the target cells. Following CAR T-cell therapy, the antigen-negative subclones within a heterogeneous tumour population can be selected and expanded by immune pressure followed by additional clonal evolution. The frequency and impact of antigen loss varies depending on the target antigen, disease entity, and patient population. The CD19-negative relapse rate ranges from 18% to 25% for pediatric B-ALL and 7% to 9% for adult B-ALL patients [50] and between 20% and 50% for LBCL in different clinical trials/studies [47, 49] while the frequency of de facto BCMA loss in MM is largely unknown [51].

Regarding the mechanisms for CD19 resistance in B-ALL, the most common one represents mutations in exons 2–5 of the *CD19* gene [52, 53]. Another mechanism is lineage switch from B-ALL to acute myeloid leukaemia, which has been observed in approximately 7% of patients [54]. The findings regarding CD19 resistance in LBCL have so far shown that mutations in the *CD19* gene appear to be an uncommon phenomenon. A non-invasive study of a large number of relapsed/refractory LBCL patients receiving anti CD19-CAR T-cells identified that alterations of multiple classes of genes, particularly those defining B-cell identity (*IRF8, PAX5*), involved in immunoregulation (*CD274*), shaping immune microenvironment (*TMEM30A*) and in *TP53*, were associated with resistance [55], whereas somatic mutation mediated CD19 loss was a relatively uncommon mechanism of resistance. The only one comparable tissue-based study analysed sequential samples from nine LBCL patients prior and post anti-CD19 CAR T-cells. While the histopathologic features were mostly retained at relapse in 7/9 patients with the exception of frequent loss of one or several B-cell-characteristic molecules, the remaining two cases exhibited a dramatic phenotypic shift with reduction of B-cell-characteristic molecules and emergence either of T-cell, or histiocytic markers [56]. None of the post CAR T-therapy samples harboured *CD19* gene mutations, while new pathogenic variants were acquired after therapy including mutations triggering the PI3K pathway or associated with tumor aggressiveness (*KRAS*, *INPP4B*, *SF3B1*, *SYNE1*, *TBXL1XR1*). A further resistance mechanism identified in that study is the occurrence of transdifferentiation in two cases as mentioned above. Both cases had similar cytogenetic abnormalities as the original LBCL with similar IGH rearrangements indicating clonal relatedness. The in-depth analysed post CAR T tumour with aberrant T-cell phenotype showed an acquired *PAX5* mutation and reduced mRNA expression of most B-cell genes with increased methylation of their promoter. There are also two additional case reports on transdifferentiation of B-cell neoplasms after CAR T-cell therapy into either a sarcoma or a poorly differentiated neoplasm [57, 58]. Both reported on similar findings as Laurent et al. [56] by showing an acquisition of gene mutations post CAR T-cell therapy with subsets not usually seen in lymphoid malignancies and in the case of sarcoma a hypermethylation and silencing of the B-cell programming genes. Regarding the mechanism for resistance in MM, several reports demonstrated a rare occurrence of a homozygous *BCMA* gene deletion resulting in loss of BCMA expression [59, 60]

**Fig. 5:**
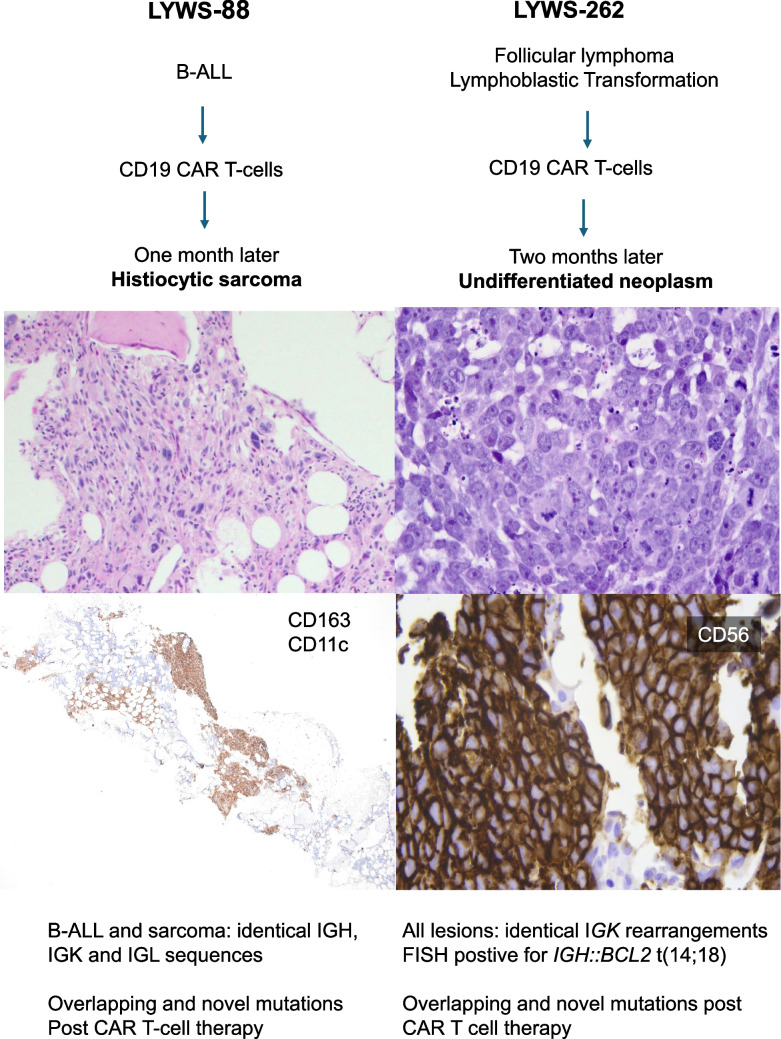
Transdifferentiation in CAR T-cell treated B-cell lymphomas. Overview of the histological, immunophenotypic and molecular findings of LYWS-88 submitted by E. Fenu and LYWS-262 (images courtesy of A. Torabi) with resistance to anti CD19 CAR T-cell therapy due to transdifferentiation of treated neoplasms

The two workshop cases fitting to CAR T-cell therapy resistance (LYWS-88 submitted by E. Fenu, and LYWS-262 presented by A. Torabi) further expand our knowledge regarding transdifferentiation as an escape mechanism from immunotherapy pressure (Figure 5). The first case regards an 8-year-old female with B-ALL that relapsed multiple times despite treatment by two different protocols and a matched sibling bone marrow transplant (BMT). She received CD19-targeting CAR T-cell therapy followed by a second unrelated BMT but remained MRD-positive and developed extramedullary lesions, which were treated with a PD-1 inhibitor. One month after CAR T-cell infusion, a BM biopsy and flow cytometry were negative for B-ALL, but there was a minute focus of atypical spindled cells within the periosteum. A subsequent BM biopsy showed an infiltrate of pleomorphic cells with irregular nuclei, prominent nucleoli, and ample cytoplasm. The cells expressed CD68, CD163, and CD11c leading to the diagnosis of histiocytic sarcoma. NGS analysis detected the same IGH, IGK, and IGL sequences as the B-ALL proving clonal relatedness. In addition, comparative analysis with the B-ALL revealed the acquisition of new mutations in the histiocytic neoplasm thus confirming transdifferentiation of B-ALL to a histiocytic sarcoma. This is apparently the first case of B-ALL that showed a transdifferentiation to a sarcoma. The second case was a man in early 50 s initially diagnosed with a follicular lymphoma grade 1–2 in a lymph node biopsy and DLBCL in the staging BM biopsy. After DA-EPOCH-R chemotherapy, a lymphoblastic transformation of the lymphoma was observed. Due to critical disease progression, he received anti-CD19 CAR T-cells. After an initial response, PET/CT scan showed multiple bone and soft tissue tumours. Biopsies showed a small round blue cell tumour that only expressed CD56 and p53 while being negative for an array of B-cell- and T-cell markers as well as for TdT markers for epithelial and neuroendocrine differentiation. FISH identified the presence of the *IGH::BCL2* that was also present in all lymphoma manifestations prior to CAR T-cell therapy and NGS identified acquisition of gene mutations when compared with the specimens prior to therapy, while some mutations were no longer detectable. Interestingly, *CD19* and *PAX5* genes were intact in this case. This case thus represents a transdifferentiated transformed follicular lymphoma following CAR T-cell therapy.

### Secondary T-cell lymphomas after CAR T-cell therapy

This main potential long-term risk is currently under the US-Food and Drug Administration (FDA) scrutiny [61] documented by the report of a “Serious Risk of T-cell malignancy following BCMA-Directed or CD19-Directed Autologous CAR T-Cell Immunotherapies”. Notably, this report suggests that some of the T-cell lymphoma cases after CAR T were “CAR-positive” highlighting a potential association between CAR transduction and transformation. Available data suggest that the risk of a second primary malignancy is not higher than expected among patients with substantial previous exposure to chemotherapy [62]. The 20 to 25 reported cases of T-cell lymphoma are, however, attracting particular interest due to the possibility of a direct causal link to CAR T-cell therapy. Among the cases for which adequate data are available, the lymphomas manifested within 2 years post-infusion. In cases for which genetic sequencing has been performed, the CAR transgene has been detected in the malignant clone, which indicates a direct causal link [61, 63–65].

**Fig. 6:**
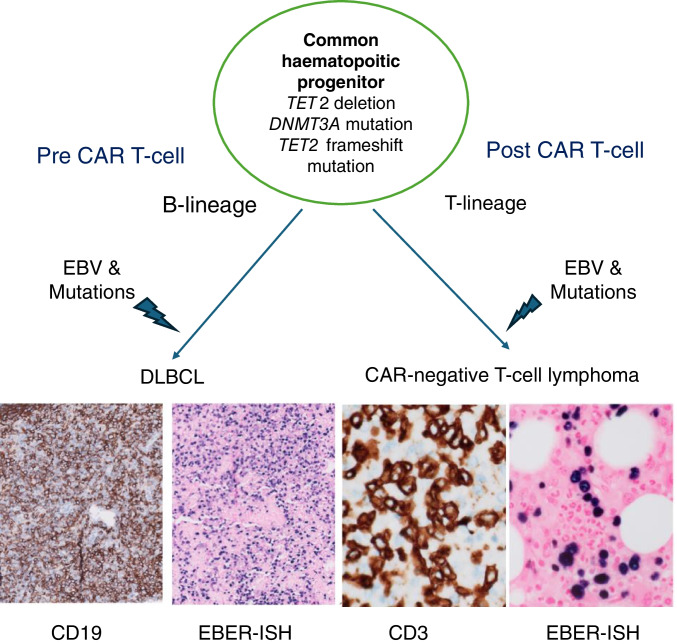
Secondary T-cell lymphoma after CAR T-cell therapy of a B-cell lymphoma. Schematic presentation of the findings in case LYWS-172 (images courtesy of P. L. Bulterys) illustrating the parallel development of EBV-positive diffuse large B-cell lymphoma and EBV-positive T-cell lymphoma from a common haematopoietic progenitor cell population

Case LYWS-172 presented by D. Gratzinger revealed another scenario that can lead to a secondary T-cell lymphoma after CAR T-cells, namely that the harvested T-cells already harbour cancer associated variants (Figure 6). The patient, a 59-year-old woman with a history of psoriasis and eosinophilic fasciitis treated with steroids, methotrexate, and mycophenolate-mofetil, developed an EBV+ DLBCL. As R-CHOP and RGemOx chemotherapy led only to an incomplete response, the patient received CD19 CAR T-cell therapy. One month post infusion pancytopenia, increasing EBV titre and clinical findings consistent with HLH, prompted a BM biopsy. This showed an infiltration by large T-cells that were EBV+ in the EBER in situ hybridization and exhibited an aberrant T-cell immunophenotype (CD2+, CD3+/-, CD7+, CD5-, CD4+, TCR silent, CD56-, CD57-), while no expression of B-cell-characteristic antigens was detectable. The patient was diagnosed with an EBV+ T-cell lymphoma associated with HLH. PET-CT showed widespread nodal and BM disease burden and the patient passed away from disease shortly thereafter. To clarify a possible CAR T-cell derived lymphoma, several investigations were performed (flow cytometry profiling of the bone marrow specimen for surface CAR protein, single cell RNA-seq for CAR vector transgene RNA, quantitative PCR for detection of integrated CAR vector DNA and overlapping 120 bp probes against the entire CAR vector) all leading to negative results. A possible transdifferentiation of DLBCL to T-lineage was excluded by comparison of clonal IG and TCR rearrangements, chromosomal abnormalities and acquired somatic mutations. Then the question was addressed, whether a clinically occult T-cell lymphoma had been unmasked by CAR T therapy of DLBCL. Review of the original biopsies failed to identify a cytologically abnormal T-cell population; the T cells in the background exhibited a low proliferative activity and the dominant clonotype of the T-cell lymphoma was detectable at an extremely low level in the DLBCL biopsy specimen, well below the level of routine detection or overt lymphoma. Single-cell DNA sequencing data indicated that the founder population of DLBCL and T-cell lymphoma was present in the haematopoietic stem cells, which contained mutations in *TET2* and *DNMT3A* and propagated them into the common lymphoid and myeloid progenitors. EBV activation due to immune suppression was probably a common event in both lymphomas but one that led to unique subsequent mutations and structural variations that ultimately resulted in the mature neoplasms [66]. An obvious question is whether clonal haematopoiesis that is rather common in the age group of patients undergoing CAR therapy poses a critical risk factor for development of lymphoma. As thousands of CAR recipients that must have harboured pre-existing clones have not developed lymphoma, there is considerable reassurance that there is no substantial risk for secondary haematolymphoid malignancies.

### Unusual T-cell proliferations after CAR T-cell therapy

The first case (LYWS-93, submitted by P. Kaur) could not be classified by the review panel. The patient, a 31-year-old male with a transformed follicular lymphoma and multiple osseous lesions, received CD19 CAR T cells and presented 6 months later with a large left lower extremity 20 cm mass. Biopsy showed a diffuse infiltration of skeletal muscle by highly proliferative (Ki67 80%) small T cells (CD3+, CD2+, CD5+, CD8+). The mass resolved spontaneously 3 months later. As no tissue was available for further studies, one could only speculate whether it represented a delayed CAR T-cell response or an unusual T-cell lymphoproliferative disorder. The second case (LYWS-186, submitted by J. Harris) was a 74-year-old man with relapsed/refractory MM who developed 2 weeks post-BCMA CAR T cell infusion hyperleucocytosis with absolute lymphocytosis and 2 months post-infusion with an altered mental status. Peripheral blood and cerebrospinal fluid contained CD4+ T cells with variable CD7 expression without evidence of clonality. Flow cytometry disclosed that they represented an unusually sustained CAR T-cell expansion. Ongoing diarrhea (3.5 months post-infusion) prompted GI biopsies showing absent plasma cells and chronic epithelial injury mimicking CVID. RNA-scope disclosed that the T cells in the lamina propria also corresponded to anti-BCMA CAR T cells, thus representing another case of the rarely observed immune effector cell-associated enterocolitis [67]. It might be possible that eradication of BCMA-positive long-lived IgA plasma cells in gut mucosa is involved in pathogenesis. Both cases underline the need for reliable and standardized methods to detect CAR T cells in pathology laboratories. Table 2 presents an overview for various currently available techniques.

**Table 2:** Summary of the currently available methods for detection of CAR T cells in pathology laboratories (adapted from Reference [68])

Method	Target	Advantages	Disadvantages
Flow cytometry	Detection of CAR-protein on cell surface: Protein L; anti-idiotype antibody; anti-linker antibodies; antigen Fc.	• High throughput • Multiplexing • Phenotype assessment	• Lack of spatial localization • Availability and specificity of antibodies • Variability of staining and gating protocols • Difficulty detecting low-frequency CAR T-cells
Immunohistochemistry	Anti-linker antibodies against CD19 CAR; Anti-EGFR against truncated EGFR tag; antibodies against specific epitopes within CAR	• Spatial localization • Multiplexing	• Availability and specificity of antibodies • Difficulty to be quantitiative
In situ hybridization	RNA sequences in CAR transcript (i.e. woodchuck hepatitis posttranscriptional regulating element)	• Spatial localization • Multiplexing	• Dependent on RNA stability

*CAR* chimeric antigen receptors; EGFR: epidermal growth factor receptor

## Complications of SLL/CLL treated by Bruton tyrosine kinase inhibitors

Bruton tyrosin kinase inhibitors (BTKIs) are being widely used as an efficacious therapy for patients with small lymphocytic lymphoma/chronic lymphocytic leukemia (SLL/CLL). Such medication is administered as continuous therapy until unacceptable toxicity or disease progression/transformation occur. Richter transformation of CLL/SLL into aggressive lymphoma occurs in 2%–10% of patients [69] and has been described as an early event in ibrutinib-treated patients [70, 71]. DLBCL is the most common type of Richter transformation and has a poor prognosis with an overall survival of 12 months [72]. Approximately 25% of the patients who discontinued BTKI therapy experienced rapid disease progression [73]. Besides discontinuation of BTKI therapy, various side effects of this drug type including bleeding, various toxicities as well as drug-drug interactions may necessitate dose modifications or interruption of treatment. BTKI-treatment is also typically interrupted for 3–7 days before and after surgery to reduce bleeding risk as well as in case of serious infection. The few reports dealing with temporal interruption of BTKI treatment mostly due to drug unrelated causes described also a disease flare in 25% of the patients as reported after treatment discontinuation. This disease flare manifested as clinical symptoms, laboratory abnormalities, or radiographic findings of progressive disease [74]. Symptoms/signs in these cases promptly resolved after drug reinitiation. There was no pathological assessment of these cases and the sole predictive factors were progressive disease at time of hold and more than 24 months of BTKI exposure.

In contrast to the aggressive presentation of Richter transformation, there is a condition known as “pseudo-Richter” transformation representing an incidental finding in SLL/CLL patients with temporary interruption of BTKIs due to surgery/acute infection [75, 76]. Up to now, eight reported patients exhibited features of histologic transformation with DLBCL-like morphology with CLL-like and non-GCB (Hans) phenotype, were frequently IGHV mutated (5 tested), exhibited trisomy 12 (6/8), *TP53* mutation/17p deletion (5/8), and no *MYC* rearrangement, whereas none showed latent EBV infection by EBER in situ hybridization. Testing for mutations in *BTK* or its immediate downstream target *PLCG2* was not performed in any of these cases. None of the cases exhibited progressive/transformed disease after resumption of BTKI treatment (3–32 months follow-up). In contrast, true Richter transformation is refractory to BTKI and requires aggressive chemotherapy.

**Fig. 7:**
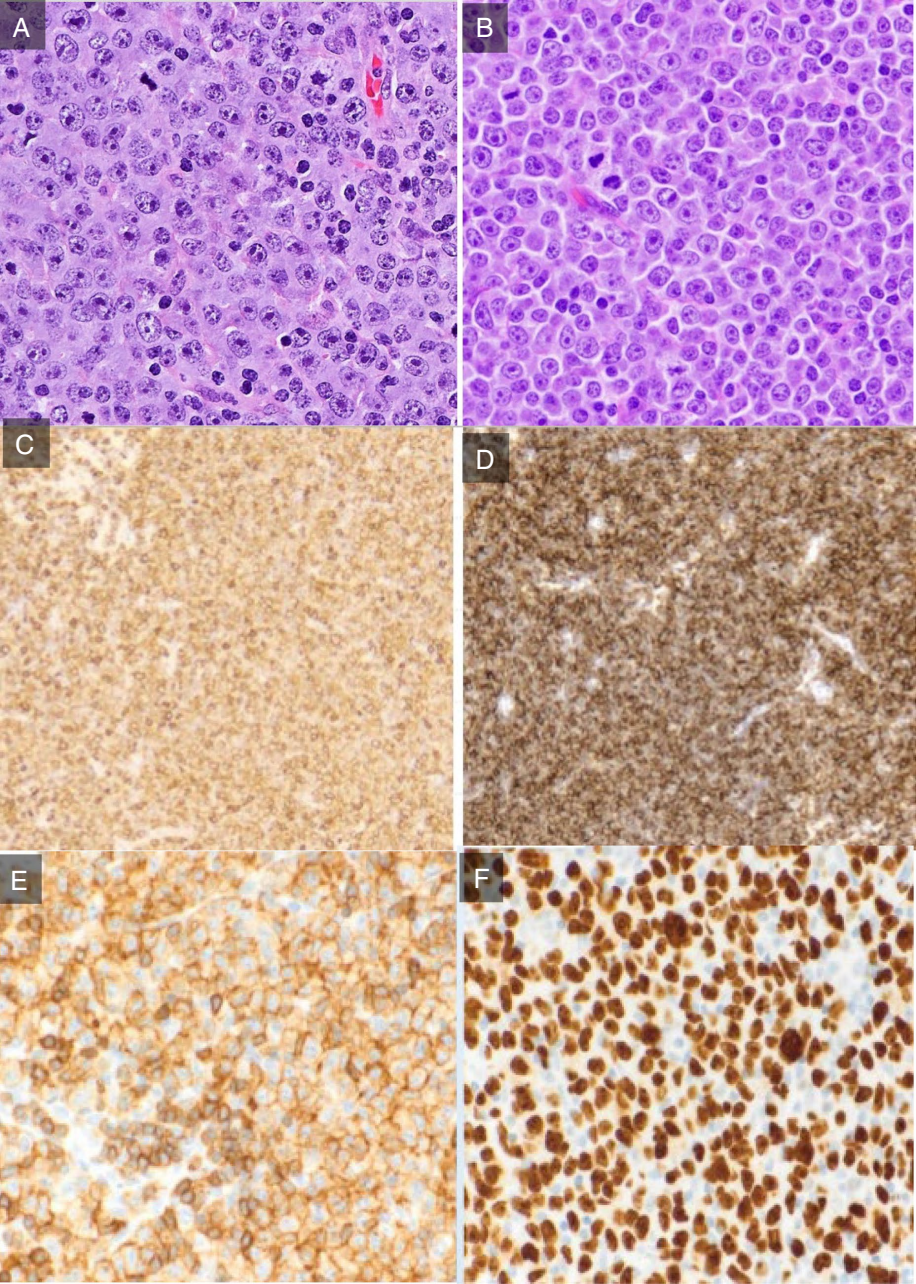
Pseudo-Richter transformation. Morphology and immunophenotypic findings in pseudo-Richter transformation. The infiltrate is composed of sheets from cells with centroblastoid and immunoblastic features (A LYWS-26 courtesy of P. Farinha; B LYWS-221 courtesy of L. Qiu) usually co-expressing CD20 (C, LYWS-221), CD23 (D, LYWS-221), and CD5 (E, LYWS 90-2 courtesy of B. Shah) and show high Ki67 index (F, LYWS-90-2 courtesy of B. Shah)

The 6 cases submitted to this workshop section (LYWS-26 presented by P. Farinha, LYWS-90-1 and 90-2 submitted by B. Shah, LYWS-97 submitted by K. Kamarádowá, LYWS-218 submitted by G. Lukose and LYWS-211 submitted by L. Qiu) had in common that an incidental histological examination of lymph nodes for detected or clinically suspected concomitant malignancy was performed. In all cases with reported history, BTKI treatment was interrupted 5–7 days prior to surgery (Supplemental Table 6). The investigated lymph nodes did not exhibit infiltrates of the suspected malignancies but showed either a vaguely nodular or diffuse infiltrate composed of sheets of cells with centroblastoid or immunoblastic features occasionally admixed with some prolymphocytes and paraimmunoblasts. All cases retained the CLL/SLL immunophenotype with co-expression of CD20, CD23, and CD5 with variable intensities with the exception of one case (LYWS-90-1) where CD5 was almost absent (Figure 7). With the exception of case LYWS-90-1, all other cases showed an additional expression of IRF4/MUM1. In two of these cases (LYWS-90-1 and LYWS-221), the peripheral blood also exhibited circulating suspicious blastoid cells. FISH analysis for *MYC* or for additional *BCL2* and *BCL6* translocations was done in three cases (LYWS-90-1, 90-2, and LYWS-221) with negative results. Only in one case (LYWS-218), a comparative study with the CLL in peripheral blood was performed that showed a novel acquisition of *TP53* mutation in the “pseudo-Richter” sample. Unfortunately, the observation period of this patient is still too short to permit further conclusions.

In all cases, there was no disease progression after reinitiation of BTKI treatment. Interestingly in case LYWS-90-1 BTKI hold lasted for 2.5 years due to postoperative complications without any disease progression. Awareness of pseudo-Richter transformation is thus critical to avoid not only a premature cessation of effective BTKI therapy but also an increased patient morbidity with aggressive chemotherapy intended for true Richter transformation.

One additional CLL case treated with BTK inhibitor ibrutinib for 3 years and 10 months (LYWS-17 submitted by A. Tzankov) developed a tumorous mass obstructing the left bronchus. This mass exhibited histologic and immunophenotypic features of an ALK-negative anaplastic large cell lymphoma. This is the second case of a T-cell lymphoma observed by the authors to occur in a CLL patient treated with ibrutinib. As there is evidence that ibrutinib interferes with T-cell functions [77–79], it may be hypothesized that this treatment might increase the risk of developing T-cell lymphoma.

## Dasatinib-associated lymphadenopathy

Dasatinib is a second-generation oral ABL tyrosine kinase inhibitor (TKI) that has been approved for treatment of chronic myeloid leukemia (CML) and Ph+ B-ALL. Besides ABL, it inhibits among many targets also PDGFR-β, C-KIT, and SRC tyrosine kinases and can suppress memory T-cell function. There is a spectrum of lymphoid proliferations reported to be associated with dasatinib treatment: (i) a transient peripheral lymphocytosis composed of T- and NK LGLS secondary to splenic contraction [80], (ii) pleural and pericardial effusions in about 30% of the patients [81] with the rare observation (6 cases reported) of HHV8- and EBV-negative primary effusion-based lymphoma (ICC)/fluid overload-associated large B-cell lymphoma, EBV-positive or EBV-negative, iatrogenic setting (5^th^ edition WHO) showing a benign clinical course following discontinuation of dasatinib [82], and (iii) lymphadenopathy as described in the next paragraph.

Up to now, 18 cases of lymphadenopathy associated with dasatinib have been reported in the literature, all in the setting of CML [83–87]. All published cases displayed a predominant follicular hyperplasia, sometimes accompanied by interfollicular/paracortical hyperplasia. The usually florid follicular hyperplasia showed large germinal centers with irregular borders and occasionally broad invaginated mantle zones arranged as cuffs surrounding perforating capillaries and thus exhibited hybrid features of progressive transformation of germinal centers and Castleman-type changes. In two cases an EBV-reactivation could be identified with EBER-positive cells within germinal centers and in interfollicular areas [23, 84]. Dasatinib treatment was discontinued leading to a regression of lymphadenopathies in the patients with available follow up (range 2–9 weeks). Dasatinib was replaced in most patients with another tyrosine kinase inhibitor with no reported recurrence of lymphadenopathy. The mechanism of dasatinib-associated lymphadenopathy is unknown. The risk of malignant transformation is currently also unknown with some reports in the literature of B-cell lymphoma development associated with dasatinib and bosutinib treatment.

**Fig. 8:**
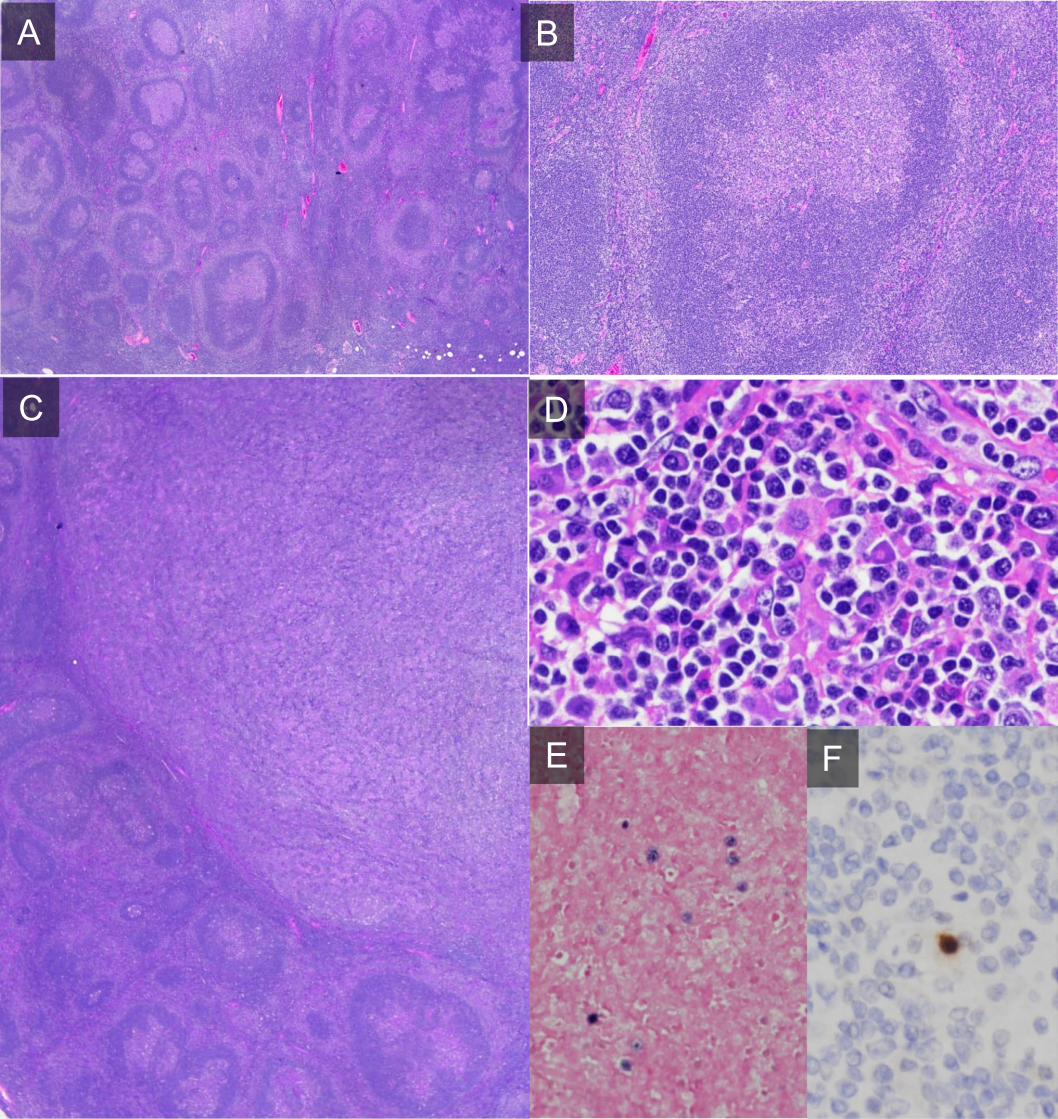
Dasatinib associated lymphadenopathy. Case LYWS-226 (images courtesy of M. Movassaghi) showed in large areas the typical picture of a florid follicular hyperplasia with irregular shaped follicles and focal progressive transformation of germinal centers (**A** overview and **B** detail of an irregular shaped follicle). There was also a demarcated area (**C**) with a polymorphic infiltrate composed of small lymphocytes, scattered B-immunoblasts, and plasma cells (**D**) with polytypic expression of the immunoglobulin light chains (not shown). Some immunoblasts were latently infected by the Epstein-Barr-virus (EBV) as demonstrated in EBER in-situ hybridization (**E**). In addition, there were single cells expressing the BZLF1-Protein of EBV indicative of a transition of the latent into the lytic viral infection phase (**F**)

Three cases of dasatinib-associated lymphadenopathy were submitted to this workshop session (LYWS-226 presented by I. Siddiqi, LYWS-291 submitted by M. Donzel and LYWS-188 submitted by L. B. Smith (Supplemental Table 7). All were adults (45–66 years old) with localized cervical (2) and axillary (1) lymphadenopathy. Case LYWS-226 showed a florid follicular hyperplasia as already described in the literature. There was also an accompanying EBV+ polymorphic LPD with detection of a clonal TR (Figure 8). The other case, LYWS-291, displayed a paracortical hyperplasia. The third case, LYWS-188, had an interesting clinical history with development of cervical lymphadenopathy under dasatinib that showed follicular hyperplasia in a biopsy (not submitted). Dasatinib was discontinued and the lymphadenopathy regressed after 1 month. TKI therapy was reinitiated using bosutinib that led to the development of neck lymphadenopathy. The lymph node exhibited serpiginous and irregular follicles arranged back to back with incomplete mantle zones. As flow cytometry detected a lambda-restricted CD10+ B-cell population and array CGH a loss of 3.47 Mb at 1p36.32p36.31, the review panel felt that these data were sufficient for diagnosing pediatric-type follicular lymphoma. Discontinuation of bosutinib led to disappearance of lymphadenopathy similar to a single reported case of a follicular lymphoma that disappeared after discontinuation of therapy [88].

## COVID-19 vaccination associated lymphadenopathy

Mass vaccination against coronavirus disease 2019 (COVID-19) has been a global health strategy to control the COVID-19 pandemic. mRNA-based vaccinations (Pfizer/BioNTech, New York, NY, USA, BNT162b2 and Moderna, Cambridge, MA USA, mRNA-1273) and viral recombinant vaccine (AstraZeneca, Cambridge, UK, ChAdOx1) have been proven to be successful in this respect. With the wide use of vaccines some adverse events post COVID-19 vaccinations have been reported.

Vaccine-associated lymphadenopathy (VAL) due to reactive lymph node changes is well described and reported in association with BCG, hepatitis B, HPV, and tetanus vaccines among others. This is however the first time that mRNA vaccines have been approved for clinical application. The COVID-19 vaccines act primarily through delivery of an mRNA into cells, where the mRNA is translated into a target protein against which the immune system will mount an immune response. Such vaccines will cause both cell-mediated (T cell) and humoral (B cell) responses resulting in increased likelihood of patients developing a lymphadenopathy. The histologic features of COVID-19-VAL are not well established as the vast majority of these lymphadenopathies are not biopsied. The patterns described include the following: atypical follicular hyperplasia with monotonous population of centroblasts, increased lymphoplasmacytic and plasmacytic cells, occasional weak BCL2 expression as well as a light chain restriction that varied between the different germinal centers [89]; extrafollicular activation of B blasts [90, 91]; necrotizing histiocytic lymphadenitis/Kikuchi-Fujimoto disease [92, 93]; Langerhans cell hyperplasia and hemophagocytosis [94]; idiopathic multicentric Castleman disease [95].

For this workshop session, four cases of this type of lymphadenopathy (Supplemental Table 8) have been submitted (LYWS-18 submitted by A.Tzankov, LYWS-85 submitted by M. Hanna, LYWS-149 submitted by I.N. Sah-Bandar and LYWS-384 submitted by J.L. Solórzano-Rendón). In contrast to the reported more frequent occurrence in females, most patients (3/4) were males. According to the available clinical data, the lymphadenopathy occurred 7 days up to 1 month after vaccination and in one case was apparently persistent for 6 months prior to biopsy. In two patients, there was a cervical and in the other two a generalized lymphadenopathy accompanied by splenomegaly. Only one patient exhibited a complicated clinical picture (LWS-149). This patient had a history of long-term medication with allopurinol and presented with altered mental status, fever, rash, fatigue, anemia, thrombocytopenia, and cervical lymphadenopathy. He had received a COVID-19 vaccine 7 days prior admission. In conjunction with the findings of a skin biopsy, a DRESS syndrome was diagnosed. The most frequent histological finding was a paracortical hyperplasia in three cases. These showed a hyperplasia of extrafollicular B-blasts which in one case coexisted with hyperplastic follicles exhibiting light chain restriction. One case also exhibited a population of immature myeloid cells and a marginal zone hyperplasia. The case with DRESS was characterized by features of an EBV reactivation with necrotic foci, few EBER-positive blastoid cells, single cells expressing the BZLF1 protein of EBV, and a mixture of polytypic plasma cells and eosinophils. As DRESS has been occasionally reported following COVID-19 vaccination [96–98], the review panel felt that rather the vaccination and not the long-term administration of allopurinol was responsible for DRESS in this case.

## Miscellaneous

Case LYWS-72 submitted by S-B Ng was a 46-year-old female with an intrauterine device inserted 2 years earlier. Endometrial biopsy showed an ulceration and a superficial dense B-cell predominant infiltrate without evidence of clonality that was classified as a reactive lymphoid hyperplasia. Case LYWS-138 submitted by T. Lakic was a 35-year-old male with a history of HIV infection on retroviral therapy who developed an EBV-positive lymphoma that could not further be classified by the review panel.

## Conclusions

 The cases in session 3 highlighted the importance of having sufficient clinical information including drug history and distribution of disease, which are essential for reliable diagnosis. Besides sufficient clinical information extensive molecular testing may be necessary for diagnosis. Small biopsy samples may hamper precise diagnosis in such cases (see box).

Box 1

**Table:** 

• Immunosuppressive/immunomodulatory therapy may lead to a broad spectrum of B or T/NK cell lineage LPDs
• B-lineage LPDs are usually EBV positive
• Polymorphic B lineage LPDs in this cohort were frequently associated with steroids, while monomorphic B lineage LPDs are associated with a broad variety of drugs (MTX, azathioprine, mycophenolate, IL23 inhibitors)
• LPDs of T or NK/T lineage range from non-clonal to clonal, are usually EBV-negative and associated with a broad variety of drugs (MTX, TNF-inhibitors, anti-Integrin antibody). Their diagnosis is challenging, molecular tests are necessary.
• LPDs associated with therapeutic interventions for solid tumors were extremely variable without clear-cut pathogenesis in most instances.
• Diagnosis of DRESS is problematic as the criteria in the literature remain conflicting and controversial
• DRESS-associated histologic patterns observed in lymph nodes are variable. The most difficult differential diagnosis is with T-cell lymphoma (especially TFH lymphomas) where clonality analysis is essential for diagnosis as these cases do not harbor clonal T-cell expansions.
• LPDs observed after CAR T-cell therapy for B-cell neoplasms exhibited unexpected phenotypes resulting either from lineage switching/transdifferentiation, or from harvested T cells already harboring cancer-associated variants.
• Unusual morphological/immunophenotypical findings in infiltrates post CAR T-cell therapies should prompt extensive additional studies including in-depth molecular analysis to verify their pathogenesis
• In situ detection of CAR T cells is possible, but still fraught with problems
• Pseudo-Richter transformation can only be diagnosed in conjunction of clinical data on temporal interruption of BTKI treatment in SLL/CLL patients. It resembles Richter transformation by morphology, exhibits a retained SLL/CLL immunophenotype and disappears after reintroduction of therapy
• Dasatinib-associated lymphadenopathy is a rare condition in CML patients with a characteristic morphology of florid follicular hyperplasia with large, irregular germinal centers. Clonality analysis is advisable in difficult cases. Discontinuation of treatment leads to spontaneous regression.
• COVID-19 associated lymphadenopathy has been thoroughly analysed only in a limited number of cases. Paracortical hyperplasia was the most frequent finding in the submitted cases. DRESS may occur in association with this type of vaccination.

## Supplementary Information

Below is the link to the electronic supplementary material.

Supplementary file1 (DOCX 32 KB)

## Data Availability

Not applicable
