# Supplementary material for: Atypical lymphoid proliferations associated with therapeutic intervention: a report of the 2024 EA4HP/SH lymphoma workshop
Source: Virchows Arch. 2025 Aug 13;487(2):287–307. doi: 10.1007/s00428-025-04197-0 (PMC12390900; doi:10.1007/s00428-025-04197-0)
Supplement: Supplementary file 1 — Supplementary file1 (DOCX 32 KB) [file 428_2025_4197_MOESM1_ESM.docx]

**Supplemental Table 1.** Polymorphic B-cell lymphoproliferative disorders associated with immunosuppressive / immunomodulatory therapies.

| Case | Age / Gender | Condition | Drug | Site | Panel Diagnosis | Follow-up/ Outcome |
| --- | --- | --- | --- | --- | --- | --- |
| LYWS-87 | 21 / M | Ulcerative colitis | Steroids Vedolizumab | Multiple colon lesions | WHO5: EBV+ Mucocutaneous ulcer, iatrogenic immunosuppression   ICC: Iatrogenic LPD, EBV+ polymorphic B-cell type | N/A |
| LYWS-323 | 79 / F | ITP and Breast cancer | Prednisone  Romiplostim | Lymph node | WHO5: Polymorphic LPD, EBV+, iatrogenic immunosuppression  ICC: Iatrogenic LPD, EBV+ polymorphic B-cell type | N/A |
| LYWS-453 | 67 / F | Dermatomyositis | Deflazacort | Subcutaneous | WHO5: Polymorphic LPD, EBV+, iatrogenic immunosuppression  ICC: Iatrogenic LPD, EBV+ polymorphic B-cell type | N/A |

M: male; F: female, ITP: idiopathic thrombocytopenic purpura; EBV. Epstein-Barr virus; LPD: lymphoproliferative disorder; N/A: not available

**Supplemental Table 2.** Monomorphic B-cell lymphoproliferative disorders associated with immunosuppressive / immunomodulatory therapies

| Case | Age / Gender | Condition | Drug | Site | Panel Diagnosis | Follow-up/ Outcome |
| --- | --- | --- | --- | --- | --- | --- |
| LYWS-234 | 69 / F | Eosinophilic granulomatosis with polyangiitis | Methotrexate | Lung | WHO5: Extranodal marginal zone lymphoma (with background EBV reactivation)  ICC: Extranodal marginal zone lymphoma (with background EBV reactivation) | Surveillence |
| LYWS-359 | 59 / F | Mixed connective tissue disease | Mycophenylate Hydroxychloroquine | CNS | WHO5: Diffuse large B-cell lymphoma, EBV+, immunosuppressive therapy  ICC: Iatrogenic immunodeficiency-associated LPD, EBV+ diffuse large B-cell type | N/A |
| LYWS-379 | 40 / M | Chronic plaque psoriasis | Guselkumab | Cervical lymph node | WHO5: Classic Hodgkin lymphoma, EBV+, immunomodulator therapy  ICC: Classic Hodgkin lymphoma, EBV+ (likely iatrogenic after immunomodulator therapy) | ABVD+RT |
| LYWS-388 | 71 / M | Follicular lymphoma | CHOP | Lymph node | Unable to fully classify from available material | N/A |
| LYWS-474 | 75 / F | Autoimmune hepatitis | Azathioprine | Liver, lung, bone, lymph nodes | WHO5: Diffuse large B-cell lymphoma, EBV+, immunosuppressive therapy  ICC: Iatrogenic immunodeficiency-associated LPD, EBV+ diffuse large B-cell type | Died of disease 1 month after diagnosis |

F: female; M: male; CHOP: cyclophosphamide, doxorubicin hydrochloride, vincristine sulfate, prednisone; EBV: Epstein-Barr virus; LPD: lymphoproliferative disorder; ABVD: doxorubicin, bleomycin, vinblastine, dacarbazine; RT: Rituximab; NA: not available

**Supplemental Table 3.** T- or NK-cell lymphoproliferative disorders associated with immunosuppressive / immunomodulatory therapies

| Case | Age / Gender | Condition | Drug | Site | Panel Diagnosis | Follow-up/ Outcome |
| --- | --- | --- | --- | --- | --- | --- |
| LYWS-193 | 63 / F | Rheumatoid arthritis | Methotrexate Etanercept | Lymph node | Atypical lymphoproliferative disorder of non-canonical Tfh cells | Died, two days post-biopsy |
| LYWS-124 | 70 / M | Crohn’s disease | Multiple R-CHOP | Lymph node | 1. DLBCL, EBV+, iatrogenic (WHO5) / Iatrogenic immunodeficiency-related LPD, EBV+ DLBCL type (ICC)  2. Peripheral T-cell lymphoma NOS (WHO5 and ICC) | N / A |
| LYWS-287 | 71 / M | Crohn’s disease | Vedolizumab | PB/BM | WHO5: NK-large granular lymphocyte leukemia, iatrogenic  ICC: Chronic LPD of NK-cells | None |
| LYWS-473 | 79 / M | CLL, Classic Hodgkin lymphoma | Multiple chemotherapies Everolimus | Ascites fluid | Atypical clonal T-cell proliferation | Hospice |

F: female; M: male; CLL: chronic lymphocyticleukemia; R-CHOP: rituximab-cyclophosphamide, doxorubicin hydrochloride, vincristine sulfate, prednisone; PB: peripheral blood; BM: bone marrow; LPD: lymphoproliferative disorder; DLBCL: diffuse large B cell lymphoma; NOS: not other specified;

**Supplemental Table 4.** Lymphoproliferative disorders associated with interventions for solid tumors

| Case | Age / Gender | Condition | Drug | Site | Panel Diagnosis | Follow-up/ Outcome |
| --- | --- | --- | --- | --- | --- | --- |
| LYWS-23 | 75 / F | Lung adenocarcinoma | Chemotherapy, anti-EGFR | Lymph node | WHO5: Nodal T follicular helper cell lymphoma, NOS, EBV neg, prior chemotherapy  ICC: Follicular helper T-cell lymphoma, NOS | N/A |
| LYWS-173 | 47 / F | Esophageal adenocarcinoma | Chemotherapy, radiation | Lymph node | WHO 5: Polymorphic lymphoproliferative disorder, EBV+, iatrogenic, chemotherapy  ICC: iatrogenic lymphoproliferative disorder, EBV+, polymorphic B-cell type | N/A |
| LYWS-286 | 76 / F | Melanoma | Nivolumab | Skin | Atypical T-cell proliferation, consistent with subcutaneous panniculitis-like T-cell lymphoma | N/A |
| LYWS-375 | 84 / F | Cecal adenocarcinoma | Pembrolizumab | Lymph node | WHO5: Polymorphic B-cell lymphoproliferative disorder, EBV+, pembrolizumab  ICC: Iatrogenic lymphoproliferative disorder, EBV+ polymorphic type | N/A |
| LYWS-423 | 96 / M | Melanoma Parotid FL | Talimogene laherparepvec | Skin | Clonal plasma cells at site of injection  Follicular lymphoma | N/A |

F: female; M: male; FL: follicular lymphoma; EGFR: epidermal growth factor receptor; EBV: Epstein-Barr virus; N/A: not available

**Supplemental Table 5.** Clinical and pathologic features of DRESS cases

| Case | Age / Gender | Drug | Organ dysfunction | PB Abnormalitiy | Biopsy | LN pattern | HHV6 IHC | T-cell Clonality by PCR |
| --- | --- | --- | --- | --- | --- | --- | --- | --- |
| LYWS-32 | 77 / F | Doxazocin Bendroflumethiazide | Kidney Liver | Eosinophilia (3.8x10^9^/L | Skin | N/A | ND | N/A |
| LYWS-86 | 40 / M | Thaliomide Sulfasalazine Traditional Chinese medicine | Shortness of breath Pleural effusion  Fever | Eosinophilia (3.3x109/L)  Atypical lymphocytes | LN | Paracortical hyperplasia | ND | NEG |
| LYWS-107 | 35 / F | Azithromycin | Fever | N/A | LN | AITL-like | NEG | N/A |
| LYWS-203 | 19 / F | Cephalosporin | Fever Liver | Eosinophilia | LN | Kikuchi-like | POS | NEG |
| LYWS-208 | 20 / M | Minocycline | Liver  Fever | Eosinophilia | LN | Paracortical hyperplasia | POS | NEG |
| LYWS-297 | 35 / M | Vancomycin | Liver  Hypotension  Fever | Eosinophilia | Skin and LN | AITL-like | NEG | NEG |

F: female; M: male; PB: peripheral blood; N/A not available; LN: lymph node; AITL: angioimmunoblastic T cell lymphoma

**Supplemental Table 6**.

Summary of the cinical features of the cases with the diagnosis of pseudo-Richter transformation

| Case | LYWS-26 | LYWS-90-1 | LYWS-90-2 | LYWS-97 | LYWS-218 | LYWS-221 |
| --- | --- | --- | --- | --- | --- | --- |
| Age / Gender | 72 / F | 84 / M | 58 / M | 97 / M | 73 / M | 60 / M |
| Time from CLL to BTKI | 8 years | 3years+9 months | 7years+6 months | n.a. | 2 years | n.a. |
| Time of BTKI before hold | 2 years | 14 months | n.a. | n.a. | 1 year | 1.10 years |
| Response to BTKI before hold | n.a. | n.a. | Excellent | n.a. | excellent | n.a. |
| Reason for BTKI hold | cervical lymph node dissection for melanoma | lung adenocarcinoma | prostate adenocarcinoma | cutaneous squamous cell carcinoma | coecal adenocarcinoma | suspected endometrial neoplasm |
| Duration of BTKI hold | Few days prior to surgery | 7 days prior to surgery | 5 days prior to surgery | n.a. | 7 days prior to surgery | 7 days prior to surgery |
| Treatment following BTKI hold | BTKI | BTKI but only 2.5 years later due to post-op complications | BTKI | BTKI | BTKI | BTKI |
| Response to BTKI resumption | 3 months without progression | 12 months without progression | 1 month without active disease | no progression | pending | normalization of CBC |

F female; M male; n.a. not available; BTKI Bruton tyrosine kinase inhibitor; CBC complete blood count

**Supplemental Table 7.**

Summary of the clinical and histological features of the cases with dasatinib-associated lymphadenopathy

| Case | Age / Gender | Time to onset  (months) | Lymph node location | Histological features | Course | Time to regression (months) |
| --- | --- | --- | --- | --- | --- | --- |
| LYWS-226 | 58 / F | n.a. | Axillary | Follicular hyperplasia and EBV+ polymorphic LPD | n.a. | N/A |
| LYWS-291 | 66 / M | 2 | Cervical | Paracortical hyperplasia | Discontinuation of Dasatinib, switch to Bosutinib  Spontaneous regression | N/A |
| LYWS-188 | 45 / M | n.a. | Cervical | Follicular hyperplasia  (not submitted) | Discontinuation of Dasatinib, Regression, Initiation of Busotinib | 1 |
|  |  | n.a. | Neck | Pediatric follicular lymphoma | Discontinuation of Busotinib  Regression | N/A |

F female; M male; LPD lymphoproliferative disorder; N/A not available

**Supplemental Table 8.**

Summary of the clinical and histological features of the cases with COVID-19 vaccination associated lymphadenopathy

| Case | Age / Gender | Vaccine type | Onset after vaccination | Clinical findings | Histological findings | Follow up |
| --- | --- | --- | --- | --- | --- | --- |
| LYWS-18 | 28 / M | mRNA | One month | Generalized PET+ lymphadenopathy and splenomegaly | Follicular hyperplasia with light chain restricted germinal centers and extrafollicular activation | No |
| LYWS-85 | 47 / F | mRNA | 2 weeks | Generalized lymphadenopathy and splenomegaly | Extrafollicular activation | Complete resolution after 3 months |
| LYWS-149 | 79 / M | Unknown | 7 days | Long term on allopurinol for pseudogout. Altered mental status, fever, rash, fatigue, anemia, thrombocytopenia, cervical lymphadenopathy | EBV-reactivation with necrosis | Resolution after discontinuation of allopurinol and intravenous steroids |
| LYWS-384 | 46 / M | Viral recombinant | Unknown | Persistent cervical lymphadenopathy six months after vaccination | Hyperplasia of marginal zones, paracortical hyperplasia with immature myeloid cells | Second biopsy after 5 months identical |

M male; F female
